# Supplementary material for: Modulating Glycoside Hydrolase Activity between Hydrolysis and Transfer Reactions Using an Evolutionary Approach
Source: Molecules. 2021 Oct 30;26(21):6586. doi: 10.3390/molecules26216586 (PMC8587830; doi:10.3390/molecules26216586)
Supplement: Supplementary file 1 [file molecules-26-06586-s001.zip › molecules-1412390-SI.pdf]

Supplementary Materials

Modulating Glycoside-Hydrolases Activity Between Hydrolysis and Transfer Reactions Using an Evolutionary Approach

Rodrigo Arreola-Barroso [1], Alexey Llopiz [1], Leticia Olvera [1] and Gloria Saab-Rincón [1,*]

[1] Departamento de Ingeniería Celular y Biocatálisis, Instituto de Biotecnología, Universidad Nacional Autónoma de México, Apartado Postal 510-3, Cuernavaca, Morelos, 62271, México; rodrigo.arreola@ibt.unam.mx (R.A.); alexey.llopiz@ibt.unam.mx (A.L.); leticia.olvera@ibt.unam.mx (L.O.)

* Correspondence: gloria.saab@ibt.unam.mx

Table S1. Dataset 1. Internal group: collection of PDB structures employed to determine the enrichment factors.

PDB ID	Resolution (Å)	Reported activity	Activity towards α-(1→4) bonds	CAZy subfamily	Organism	Reference
1A47	2.56	CGTase	Transferase	GH13_2	*Thermoanaerobacterium thermosulfurigenes (Clostridium thermosulfurogenes)*	[110]
1UKQ	2.00	CGTase	Transferase	GH13_2	*Bacillus sp. 1011*	[111]
2CXG	2.50	CGTase	Transferase	GH13_2	*Niallia circulans 251*	[112]
2VR5	2.80	Debranching enzyme	Transferase	GH13_11	*Saccharolobus solfataricus P2*	[113]
1MXG	1.60	α-Amylase	Hydrolase	GH13_7	*Pyrococcus woesei*	[114]
1HX0	1.38	α-Amylase	Hydrolase	GH13_24	*Sus scrofa*	[115]
1RPK	2.00	α-Amylase	Hydrolase	GH13_6	*Hordeum vulgare*	[116]
1UH3	2.60	α-Amylase	Hydrolase	GH13_21	*Thermoactinomyces vulgaris R-47*	[117]
1WPC	1.90	α-Amylase	Hydrolase	GH13_5	*Bacillus sp. 707*	[118]
2CPU	2.00	α-Amylase	Hydrolase	GH13_24	*Homo sapiens*	[119]
2WC7	2.37	Debranching enzyme	Hydrolase	GH13_20	*Nostoc punctiforme PCC 73102*	[120]
3BC9	1.35	α-Amylase	Hydrolase	GH13_5	*Halothermothrix orenii H 168*	[121]
3K8M	2.50	α-Amylase	Hydrolase	GH13_36	*Bacteroides thetaiotaomicron VPI-5482*	[122]
7TAA	1.98	α-Amylase	Hydrolase	GH13_1	*Aspergillus oryzae DSM63303*	[123]

Table S2. Dataset 2. External group: set of 3D structures used to test the ability of the enrichment factor to classify functionally enzymes in the GH13 family.

PDB ID	Resolution (Å)	Reported Activity	Activity towards α-(1→4) bonds	CAZy subfamily	Organism	Reference
1CGT	2.00	Glucanotransferase	Transferase	GH13_2	*Niallia circulans 8*	[124]
4JCL	1.70	Glucanotransferase	Transferase	GH13_2	*Paenibacillus macerans IAM1243/ IB7 / IFO 3490 (NRRL B-388) / JFB05-01 (CCTCC M203062)*	[125]
1BLI	1.90	α-Amylase	Hydrolase	GH13_5	*Bacillus licheniformis 584 / ATCC 27811*	[126]
1GCY	1.60	Maltotretrasaccharide (G4) producing α-amylase	Hydrolase	NA	*Pseudomonas stutzeri MO-19*	[127]
1WZL	2.00	Neopullunase Cyclomaltodextrinase	Hydrolase	GH13_20	*Thermoactinomyces vulgaris R-47*	[36]
AmyA	Q-mean 0.95	α-Amylase	Hydrolase	GH13_36	*Thermotoga maritima MSB8*	[128]
3BC9	1.35	α-Amylase	Hydrolase	GH13_5	*Halothermothrix orenii H 168*	[139]
3DHU	2.00	α-Amylase	Hydrolase	NA	*Lacticaseibacillus plantarum WCFS1*	[130]
3EDF	1.65	Cyclomaltodextrinase	Hydrolase	NA	*Flavobacterium sp. 92*	[131]
3VM7	2.25	α-Amylase	Hydrolase	GH13_1	*Malbranchea cinnamomea*	[132]
4AEE	2.28	Maltogenic a-amylase Cyclomaltodextrinase	Hydrolase	GH13_20	*Staphylothermus marinus F1*	[133]
4AEF	2.34	α-Amylase Cyclomaltodextrinase	Hydrolase	GH13_20	*Pyrococcus furiosus DSM 3638*	[134]
4GKL	2.40	Maltogenic α-amylase	Hydrolase	NA	*Thermotoga neapolitana DSM 4359*	[135]
4UZU	1.90	α-Amylase	Hydrolase	GH13_5	*Geobacillus stearothermophilus DY5 / PHI300 / NZ-3*	[136]

Table S3. Dataset 3. Enzymes to evaluate contact conservation and its correlation between enzymes.

PDB ID	Resolution (Å)	CAZy family	CAZy Subfamily	Organism
3VM7	2.25	GH13	1	*Malbranchea cinnamomea*
7TAA	1.98	GH13	1	*Aspergillus oryzae DSM63303*
1A47	2.56	GH13	2	*Thermoanaerobacterium thermosulfurigenes EM1*
1CGT	2.00	GH13	2	*Niallia circulans 8*
1QHO	1.70	GH13	2	*Geobacillus stearothermophilus C599*
2CXG	2.50	GH13	2	*Niallia circulans 251*
1UKQ	2.00	GH13	2	*Bacillus sp. 1011*
4JCL	1.70	GH13	2	*Paenibacillus macerans IAM1243/ IB7 / IFO 3490 (NRRL B-388) / JFB05-01 (CCTCC M203062)*
3ZSS	1.80	GH13	3	*Streptomyces coelicolor A3(2)*
1WPC	1.90	GH13	5	*Bacillus sp. 707*
1BLI	1.90	GH13	5	*Bacillus licheniformis 584 / ATCC 27811*
3BC9	1.35	GH13	5	*Halothermothrix orenii H 168*
4UZU	1.90	GH13	5	*Geobacillus stearothermophilus DY5 / PHI300 / NZ-3*
1RPK	2.00	GH13	6	*Hordeum vulgare*
1MXG	1.60	GH13	7	*Pyrococcus woesei*
3AMK	1.90	GH13	8	*Oryza sativa Japonica Group*
3AML	1.70	GH13	8	*Oryza sativa Japonica Group*
4BZY	2.75	GH13	8	*Homo sapiens*
2VR5	2.80	GH13	11	*Saccharolobus solfataricus P2*
1WZL	2.00	GH13	20	*Thermoactinomyces vulgaris R-47*
2YA0	1.85	GH13	12	*Streptococcus pneumoniae TIGR4*
3FAW	2.10	GH13	12	*Streptococcus agalactiae COH1*
2YOC	2.88	GH13	13	*Raoultella ornithinolytica 10-5246*
2FHF	1.65	GH13	13	*Klebsiella pneumoniae UNF5023*
2WC7	2.37	GH13	20	*Nostoc punctiforme PCC 73102*
4AEE	2.28	GH13	20	*Staphylothermus marinus F1*
4AEF	2.34	GH13	20	*Pyrococcus furiosus DSM 3638*
1UH3	2.60	GH13	21	*Thermoactinomyces vulgaris R-47*
1HX0	1.38	GH13	24	*Sus scrofa*
2CPU	2.00	GH13	24	*Homo sapiens*

1UA7	2.21	GH13	28	*Bacillus subtilis 2633*
1G9H	1.80	GH13	32	*Pseudoalteromonas haloplanktis TAB23 / A23*
3K8M	2.00	GH13	36	*Bacteroides thetaiotaomicron VPI-5482*
AmyA	Q-mean 0.95	GH13	36	*Thermotoga maritima MSB8*
1GCY	1.60	GH13	NA	*Pseudomonas stutzeri MO-19*
1LWJ	2.50	GH13	NA	*Thermotoga maritima MSB8*
3DHU	2.00	GH13	NA	*Lacticaseibacillus plantarum WCFS1*
3EDF	1.65	GH13	NA	*Flavobacterium sp. 92*
4E2O	2.10	GH13	NA	*Geobacillus thermoleovorans CCB_US3_UF5*
4GKL	2.40	GH13	NA	*Thermotoga neapolitana DSM 4359*
2ZQ0	1.60	GH97	NA	*Bacteroides thetaiotaomicron VPI-5482*
3W37	1.70	GH31	NA	*Beta vulgaris*

Table S4. Dataset 4: proteins reported as characterized by CAZy database [*31*]. For transferases all sequences were considered and modelled using the Swiss model if needed. For the hydrolase only sequences with reported structure were used. The ID for the structures is the PDB ID, while for models is the Uniprot or GenBank ID, followed by the PDB ID of the template used. For the models the Q-mean value is reported as resolution. All models were minimized using Rosetta.

ID (PDB, Uniprot or GenBank)	Resolution	Reported Activity in CAZy database (species)	Activity towards α-(1→4) bonds reported in CAZy	CAZy subfamily
1CDG	2.00	β-Cyclodextrin glucanotransferase (*Niallia circulans* 251)	Transferase	GH13_2
1CGT	2.00	β-Cyclodextrin glucanotransferase (*Niallia circulans 8*)	Transferase	GH13_2
1CYG	2.50	α/β-Cyclodextrin glucanotransferase (*Geobacillus stearothermophilus*)	Transferase	GH13_2
1GJU	2.40	4-α-Glucanotransferase/maltosyltransferase (*Thermotoga maritima*)	Transferase	NA
1LWJ	2.50	4- α-Glucanotransferase (*Thermotoga maritima*)	Transferase	NA
1V3M	2.00	Cyclodextrin glycosyltransferase (*Bacillus sp.* 1011)	Transferase	GH13_2
2VR5	2.80	Isoamylase / 4-α-glucanotransferase (*Saccharolobus solfataricus* P2)	Transferase	GH13_11
3BMV	1.60	α/β-Cyclodextrin glycosyl transferase (*Thermoanerobacterium thermosulfurigenes* EM1)	Transferase	GH13_2
4JCL	1.70	α-Cyclodextrin glucanotransferase (*Paenibacillus macerans* IAM1243)	Transferase	GH13_2
4JCM	1.65	**γ**-Cyclodextrin glucanotransferase (*Evansella clarkii*)	Transferase	GH13_2
A2QTS4_1cgt	QMEAN, −2.64	Cell-wall 4-α-glucanotransferase (*Aspergillus niger* CBS 513.88)	Transferase	GH13_1
A2QYT9_2aaa	QMEAN, −2.48	4-α-Glucanotransferase (*Aspergillus niger* CBS 513.88)	Transferase	GH13_1
P26827_1uks (1a47)	QMEAN, −1.85	Cyclodextrin glucanotransferase (*Thermoanaerobacterium thermosulfurigenes*)	Transferase	GH13_2
A0A077D499_3bmv	QMEAN, −0.30	Cyclodextrin glycosyl transferase (uncultured *Carboxydocella*)	Transferase	GH13_2
A0A0C4WMI5_1ciu	QMEAN, 0.72	Cyclodextrin glycosyltransferase (*Thermoanaerobacter sp.* P4)	Transferase	GH13_2
B2D1U4_1ukt	QMEAN, 0.33	Cyclodextrin glucanotransferase (*Paenibacillus sp.* JB-13)	Transferase	GH13_2
B2XY82_4jcl	QMEAN, −0.05	Cyclodextrin glycosyl transferase (*Paenibacillus graminis* MC22.13)	Transferase	GH13_2
C9WB02_1cgt	QMEAN, −0.01	β-Cyclodextrin glycosyl transferase (*Paenibacillus illinoisensis ZY-08 / ZY-8*)	Transferase	GH13_2
B1VC16_6cgt	QMEAN, 0.07	β-cyclodextrin glycosyltransferase (*Paenibacillus pabuli* US132)	Transferase	GH13_2
O82984_1ukq	QMEAN, −0.77	β-cyclodextrin glycosyltransferase (Bacillus sp. A2-5A)	Transferase	GH13_2
O86956_1lwh	QMEAN, −0.70	4-α-glucanotransferase (*Thermotoga neapolitana*)	Transferase	NA
O86959_1jf6	QMEAN, −2.52	Cyclomaltodextrinase glucanotransferase (*Thermotoga neapolitana*)	Transferase	GH13_20

P08704_4jcm	QMEAN, −3.00	α-Cyclodextrin glucanotransferase (*Klebsiella pneumoniae* M5a1)	Transferase	GH13_2
P17692_1cdg	QMEAN, 0.19	β-cyclomaltodextrin glucanotransferase (*Bacillus sp*. B1018)	Transferase	GH13_2
P31746_1pj9	QMEAN, −0.59	β-Cyclodextrin glucanotransferase (*Bacillus sp*. 1-1)	Transferase	GH13_2
P31747_6cgt	QMEAN, 0.04	Cyclodextrin glucanotransferase (*Bacillus sp*. 6.3.3)	Transferase	GH13_2
P31835_6aij	QMEAN, −0.30	α-Cyclodextrin glucanotransferase (*Paenibacillus macerans*)	Transferase	GH13_2
Q3HUR2_6aij	QMEAN, −3.55	β-cyclodextrin glucanotransferase (*Pyrococcus furiosus* DSM 3638)	Transferase	GH13_2
Q8X268_1uks	QMEAN, −1.82	β-cyclodextrin glucanotransferase (*Thermococcus kodakarensis* KOD1)	Transferase	GH13_2
Q9UWN2_1qho	QMEAN, −1.51	α-cyclodextrin glucanotransferase (*Thermococcus sp*. B1001)	Transferase	GH13_2
Q9ZAQ0_3bmv	QMEAN, −0.63	β-cyclodextrin glucanotransferase (*Geobacillus stearothermophilus* ET1)	Transferase	GH13_2
Q25CB6_4jcm	QMEAN, −0.45	g-cyclomaltdextrin glucanotransferase (*Bacillus sp.* G-825-6)	Transferase	GH13_2
Q53I75_1ukq	QMEAN, −0.07	α-cyclomaltodextrin glucanotransferase (*Haloferax mediterranei*)	Transferase	GH13_2
1AVA	1.9	α-Amylase (*Hordeum vulgare*)	Hydrolase	GH13_6
1EA9	3.20	Cyclomaltodextrinase (*Bacillus sp.)*	Hydrolase	GH13_20
1G94	1.74	α-Amylase (*Pseudoalteromonas haloplanktis*)	Hydrolase	GH13_32
1GCY	1.60	Maltotetraose-forming amylase (*Pseudomonas stutzeri*)	Hydrolase	NA
1HT6	1.50	α-Amylase (*Hordeum vulgare*)	Hydrolase	GH13_6
1HX0	1.38	α-Amylase (*Sus scrofa*)	Hydrolase	GH13_24
1J0H	1.90	Cyclomaltodextrinase / neopullulanase (*Geobacillus stearothermophilus* TRS40)	Hydrolase	GH13_20
1JAE	1.65	α-Amylase (*Tenebrio molitor*)	Hydrolase	GH13_15
1JI1	1.60	α-Amylase (*Thermoactinomyces vulgaris* R-47)	Hydrolase	GH13_21
1MWO	2.20	α-Amylase (*Pyrococcus woesei*)	Hydrolase	GH13_7
1QHO	1.70	Maltogenic α-amylase (*Geobacillus stearothermophilus* C599)	Hydrolase	GH13_2
1SMA	2.80	Maltogenic α-amylase (*Thermus sp*. IM6501)	Hydrolase	GH13_20
1SMD	1.60	α-Amylase (*Homo sapiens*, salivary)	Hydrolase	GH13_24
1UA7	2.21	α-Amylase (*Bacillus subtilis* 2633)	Hydrolase	GH13_28
1UD2	2.13	α-Amylase (*Bacillus sp*. KSM-K38)	Hydrolase	GH13_5

1VJS	1.70	α-Amylase (*Bacillus licheniformis* 584)	Hydrolase	GH13_5
1WPC	1.90	Maltohexaose-forming amylase (*Bacillus sp.* 707)	Hydrolase	GH13_5
1WZA	1.60	α-Amylase (*Halothermothrix orenii* H 168)	Hydrolase	GH13_36
2D2O	2.10	Cyclomaltodextrinase / neopullulanase (Thermoactinomyces vulgaris R-47)	Hydrolase	GH13_20
2DIE	2.10	α-Amylase (*Bacillus sp.* KSM-1378)	Hydrolase	GH13_5
2GJP	1.90	α-Amylase (*Sutcliffiella halmapala*)	Hydrolase	GH13_5
2YA0	1.85	Glycogen-degrading enzyme (*Streptococcus pneumoniae* TIGR4)	Hydrolase	GH13_12
2ZE0	2.00	α-Glucosidase (*Geobacillus sp.* HTA-462)	Hydrolase	GH13_31
3BC9	1.35	α-Amylase (*Halothermothrix orenii* H 168)	Hydrolase	GH13_5
3BH4	1.40	α-Amylase (*Bacillus amyloliquefaciens*)	Hydrolase	GH13_5
3DC0	2.78	α-Amylase (*Bacillus sp.* KR8104)	Hydrolase	GH13_28
3EDJ	1.69	Cyclomaltodextrinase (*Flavobacterium sp.* 92)	Hydrolase	NA
3VGF	2.30	α-Amylase / Maltooligosyltrehalose (*Saccharolobus solfataricus* KM1)	Hydrolase	GH13_10
3WN6	2.16	α-Amylase (*Oryza sativa* Japonica Group)	Hydrolase	GH13_6
3WY2	1.47	α-Glucosidase (*Halomonas sp.* H11)	Hydrolase	GH13_23
4AEE	2.28	Maltogenic α-amylase/cyclomaltodextrinase (*Staphylothermus marinus* F1)	Hydrolase	GH13_20
4AEF	2.34	Maltogenic α-amylase/cyclomaltodextrinase (*Pyrococcus furiosus* DSM 3638)	Hydrolase	GH13_20
4E2O	2.10	α-Amylase (*Geobacillus thermoleovorans* CCB_US3_UF5)	Hydrolase	NA
4GKL	2.40	Maltogenic α-amylase (*Thermotoga neapolitana* DSM 4359)	Hydrolase	NA
4UZU	1.90	α-Amylase (*Geobacillus stearothermophilus* DY5)	Hydrolase	GH13_5
5A2B	1.85	α-Amylase (*Anoxybacillus sp.* SK3-4)	Hydrolase	NA
5H06	1.95	α-Amylase (uncultured bacterium)	Hydrolase	GH13_37
6A0J	1.60	Cyclic maltosyl-maltose hydrolase (*Arthrobacter globiformis* M6)	Hydrolase	GH13_20
6BS6	2.17	α-Amylase / neopullulanase (*Bacteroides thetaiotaomicron* VPI-5482)	Hydrolase	GH13_36
7TAA	1.98	α-Amylase (*Aspergillus oryzae* DSM63303)	Hydrolase	GH13_1

Table S5. Enrichment factors for contact 64–65 (D98-K99 in TmAmyA). They suggested the mutations D98P/K99A to make TmAmyA (a hydrolase) more like a transglycosidase. Residues that are not in the table have an enrichment factor of zero.

**Residue contact**	**Enrichment factor by residues**						
**98**	P	C	D	A	E	T	-
	0.45	0.25	0.0	−0.1	−0.1	−0.5	-
99	A	Y	R	N	Q	M	K
	0.4	0.2	−0.05	−0.1	−0.1	−0.1	−0.3

Table S6. Enrichment values for the residues around residue 72 (F72 in TmGTase). While searching for a pair to mutate in TmGTase to augment its resemblance with a hydrolase we found this cluster of residues with high enrichment values, suggesting these residues are important for residue function. The pair of residues to mutate has an asterisk (*).

Residue Pair	Enrichment factor by residue pair							
72,88	F, I	L, G	L,L	L, V	L, A	F, L	F, V	-
	0.8	−0.09	−0.09	−0.09	−0.18	−0.18	−0.18	-
* 72,86	F, V	L,I	L, A	L,L	F, I	L, V	-	-
	0.70	0.0	−0.09	−0.18	−0.27	−0.27	-	-
72,76	F, I	F, V	F, L	L, S	L, V	L, I	-	-
	0.6	−0.07	−0.09	−0.09	−0.09	−0.76	-	-
72,175	F,W	F,F	L,F	L,Y	L, T	L, V	L,L	-
	0.50	−0.09	−0.09	−0.09	−0.09	−0.09	−0.27	-
72,73	F, Q	F, K	F, D	L, E	L, V	L, K	L, Q	F, R
	0.4	0.30	−0.09	−0.09	−0.09	−0.18	−0.18	−0.18
72,180	F, I	F, V	L,L	L, V	F, L	-	-	-
	0.30	0.12	−0.09	−0.09	−0.27	-	-	-

Table S7. Enrichment values for the residues around residue 273 (F273 in TmGTase). This trio of residues should be important to switch function as its residues have been selected both in hydrolases and transglycosidase. We mutated residues 274 and 279 (T274 and M279 in TmGTase), which are not in direct contact with the catalytic site.

Residue Pair	Enrichment factor by residue pair								
273,274	F, I	Y, V	K,L	F, L	L,L	F, V	-	-	-
	0.60	0.20	−0.09	−0.09	−0.09	−0.63	-	-	-
273,279	F, M	Y, L	F,Y	F,I	K,E	F,S	L,T	F,T	F, N
	0.60	0.20	−0.09	−0.09	−0.09	−0.09	−0.09	−0.18	−0.27

Table S1. Primers used to create the mutants used in this study.

Enzyme mutated	Mutation	Sequence (from 5′ to 3′)
TmAmyA	K98P/D99A	ACAGACTACTACAACGTCGAGCCGGCGTACGGCACCATGGAAGATCTC
TmGTase	F72L	CGATCATCTCTTTCAACTCTCTCACTACC
TmGTase	V86I	GGGAAGGTCAAGAACGATTTTTATTCCGC
TmGTase	T274V	CCCGGTGAATTTTGTTTCGAATCACG
TmGTase	M279N	CGAATCACGACAACTCGAGGCTTGCAAGC

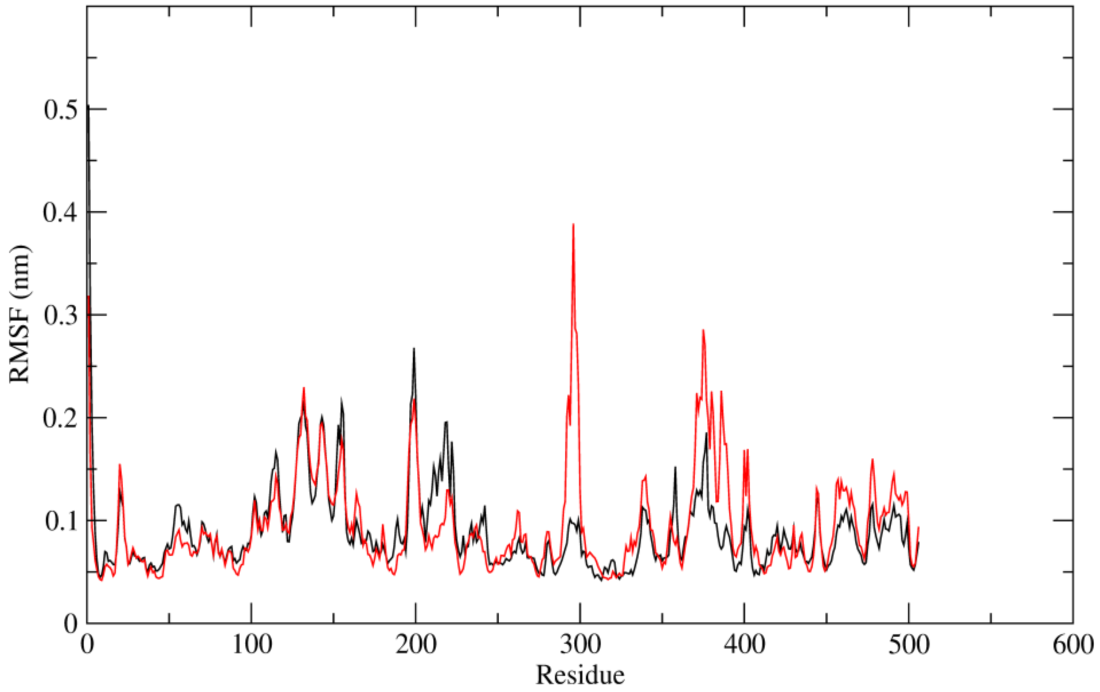

Figure S1. RMSF obtained from Molecular Dynamic (MD) simulation during 500 ns for TmAmyA wild type (red line) and K98P/D99A/H222Q mutant (black line). Here, the residue numbers are displaced by −29 relative to Liebl et al. [*36*].

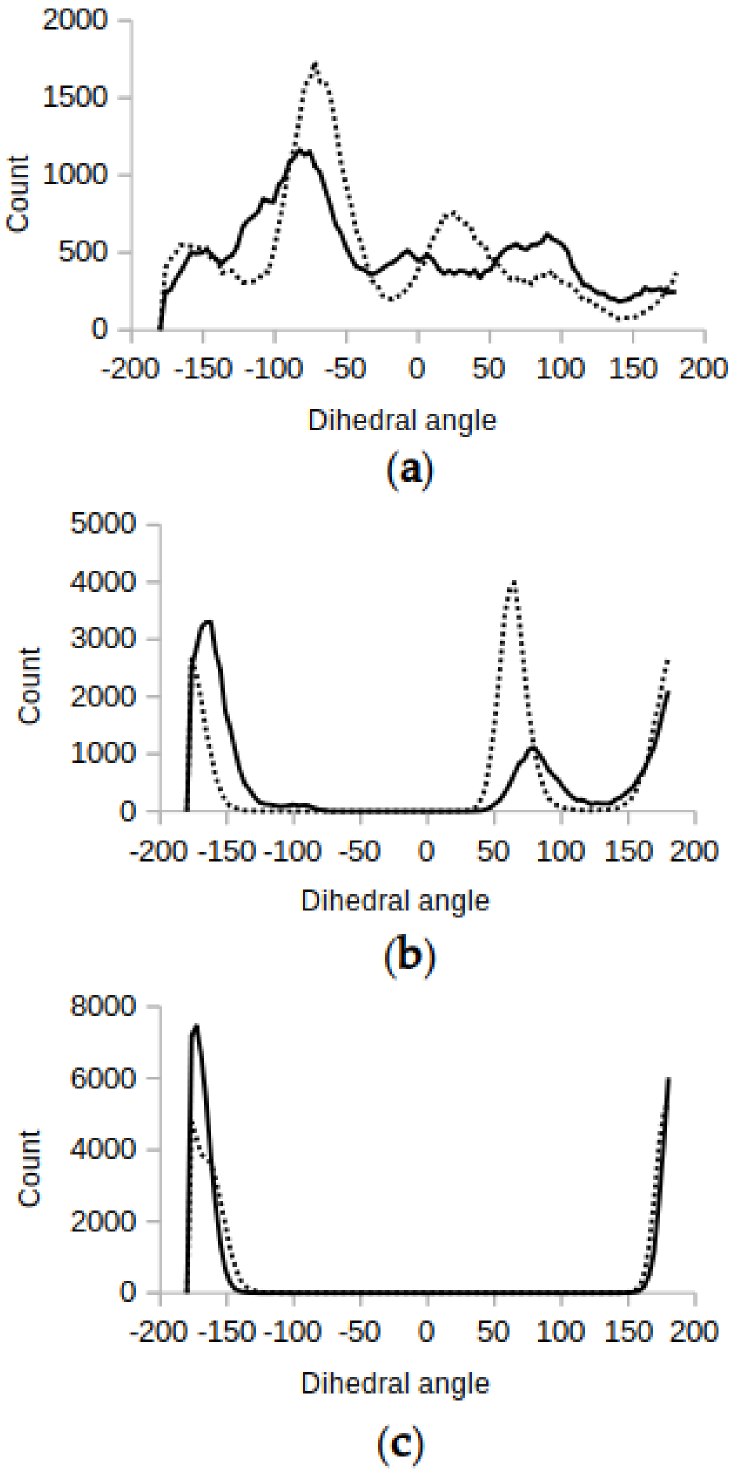

Figure S2. Conformational analysis of χ dihedral angles of acid-base residue (Glu258) of TmAmyA for wild type (continue line) and K98P/D99A/H222Q (dotted lines). (**a**) dihedral angles χ_3 (**b**) dihedral angles χ_2 (**c**) dihedral angles χ_1.

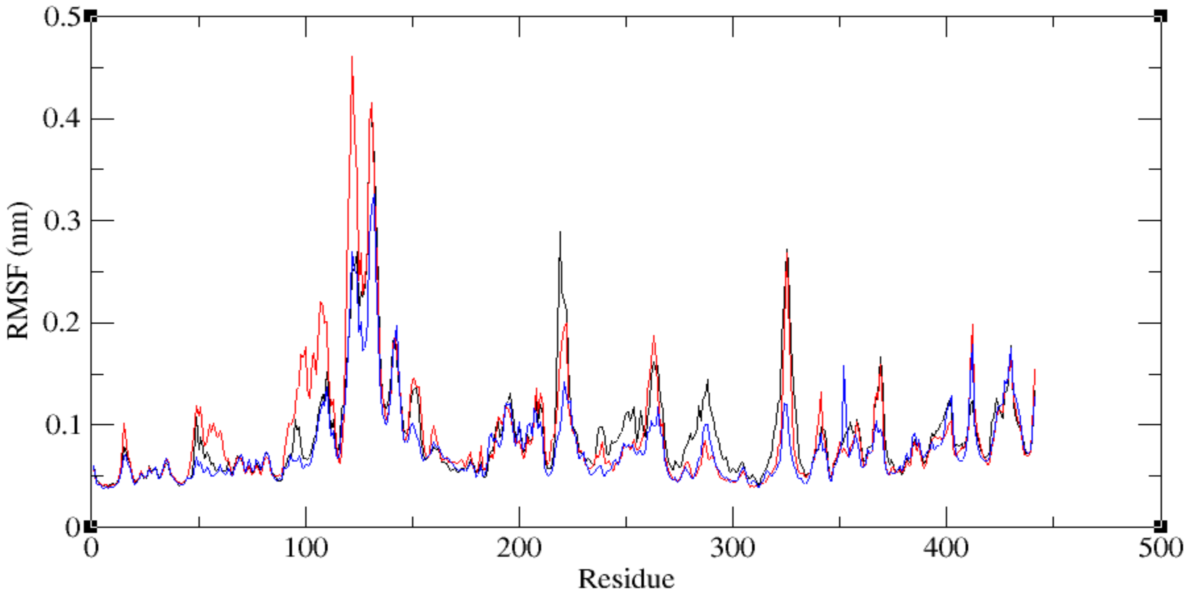

Figure S3. RMSF obtained from Molecular Dynamic (MD) simulation during 500 ns for TmGTase wild type (red line), M279N (black line) and T274V/M279N mutant (blue line).

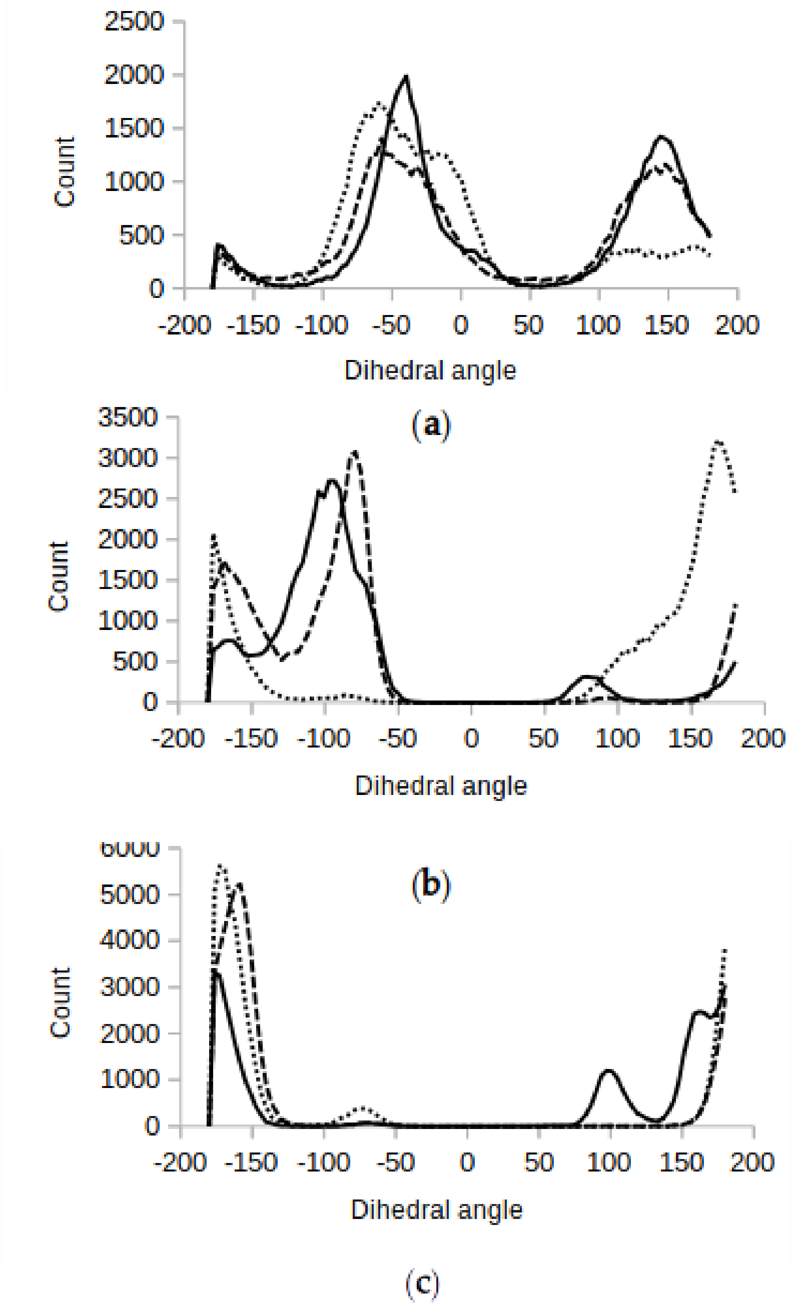

Figure S4. Conformational analysis of χ dihedral angles of acid-base residue (Glu216) of TmGTase for wild type (continue line), M279N (dashed line), and T274V/M279N (dotted lines). (**a**) dihedral angles χ_3 (**b**) dihedral angles χ_2 (**c**) dihedral angles χ_1.

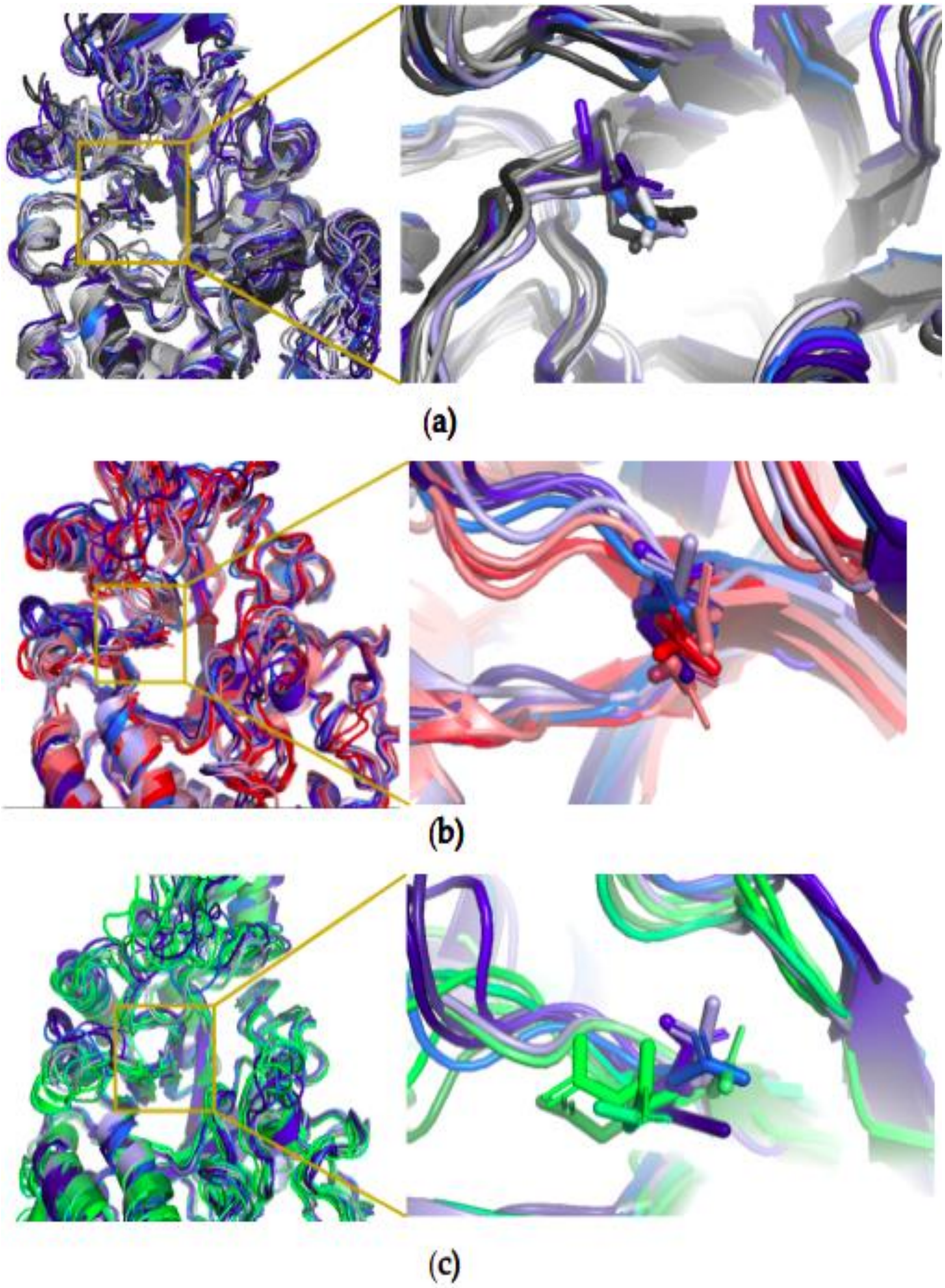

Figure S5. Representation of frames around 0.01, 100, 200, 300, and 400 ns for glycosidases. The increase in the intensity of color corresponds with the increment of frames number (**a**) Comparison of TmAmyA wild type (blue) with mutant D98P/K99A/H222Q (gray) (**b**) Comparison of TmGTase wild type (blue) with mutant T274V/M279N (red). (**c**) Comparison of TmGTase wild type (blue) with mutant M279N (green).

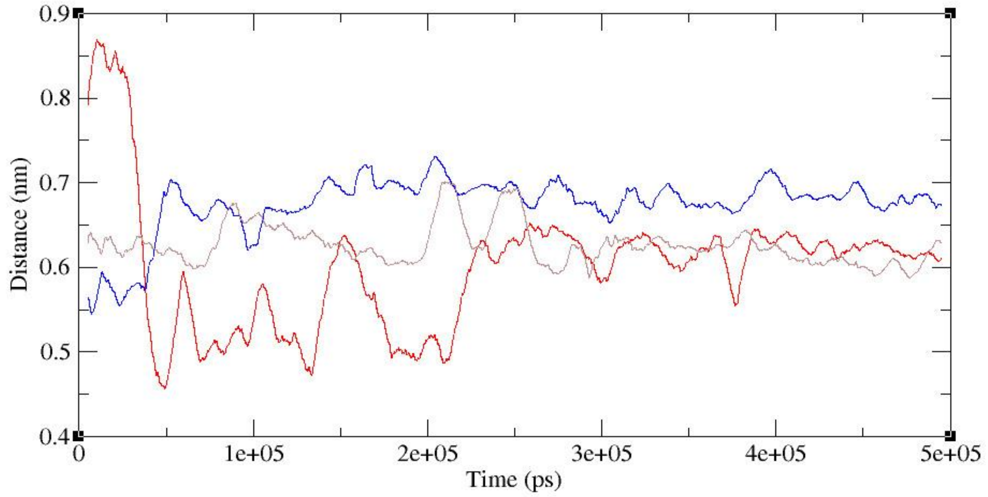

Figure S6. Change of average distance of D278 and E216 in TmGTase distance during MD simulation for wild type (blue line) M279N (red line) and T274V/M279N (gray line).

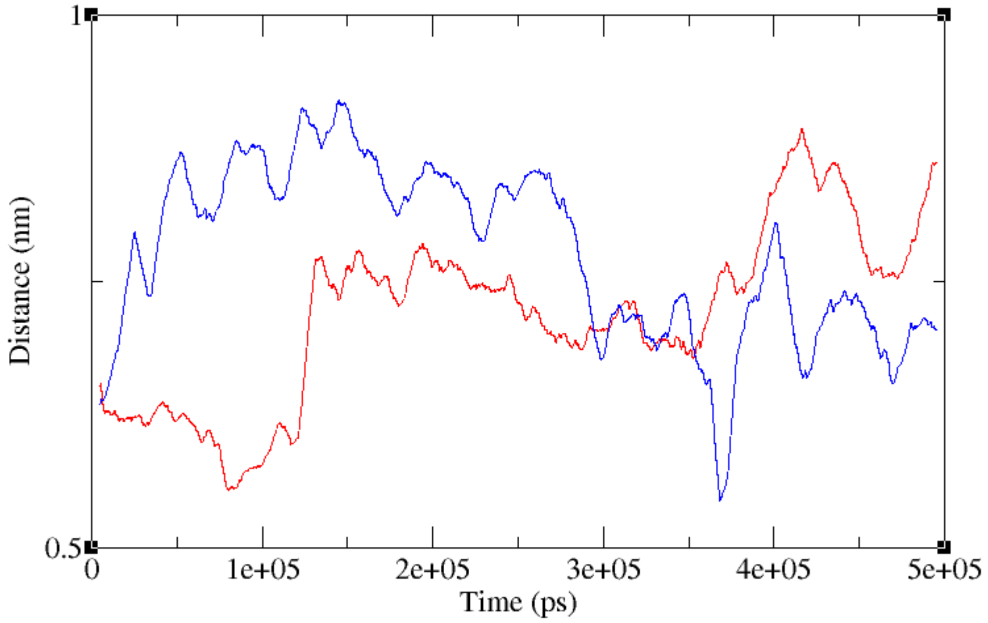

Figure S1. Change of average distance of D310 and E258 in TmAmyA distance during MD simulation for wild type (red line) and D98P/D99A/H222Q (red line).

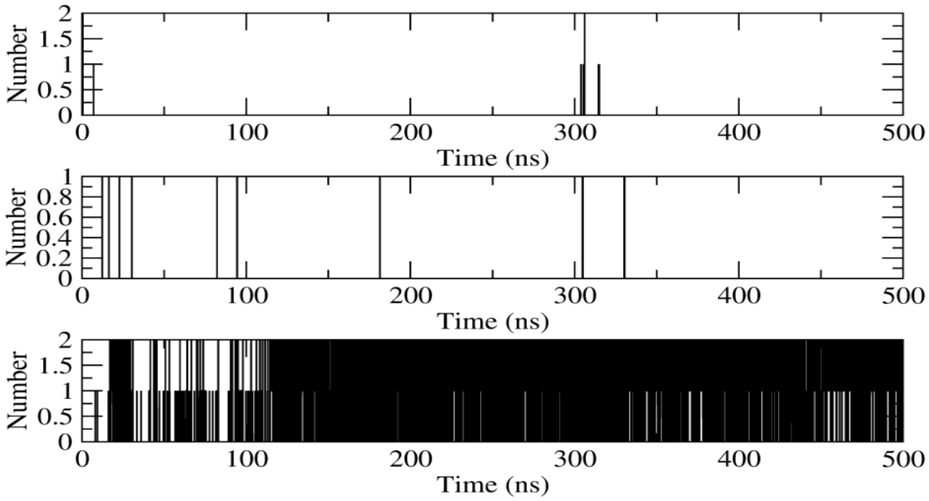

Figure S8. Number of hydrogen bonds during MD simulation of TmGTase for K324 and D278. (**a**) wild type. (**b**) M279N. (c) T274V/M279N.

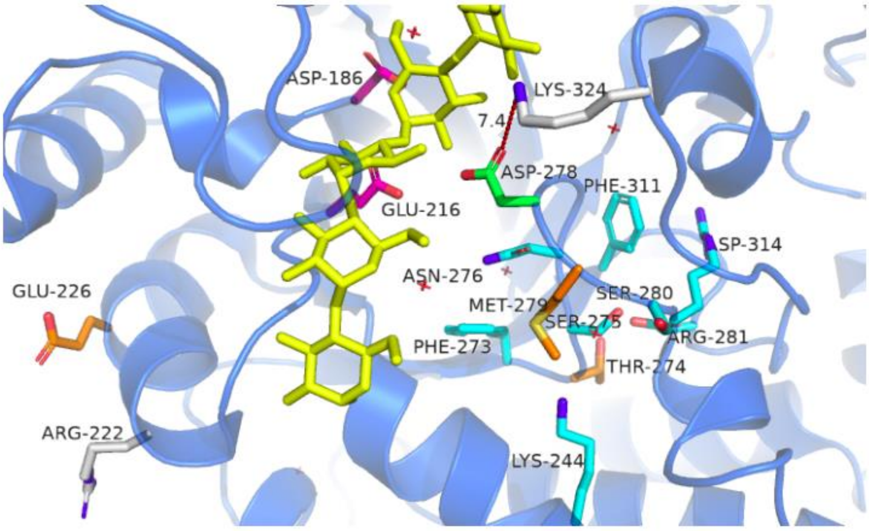

Figure S9. Structure TmGTase (PDB ID 1LWJ) highlighting the connection between the mutation sites and the catalytic residues (pink) including the binding subsites demarcated by acarbose (yellow). Residues T274 and M279 (orange sticks spheres) participate in a H-bond network (residues represented as cyan sticks). Dotted lines (red) indicate the distance between these residues. Only in T274V/M279N, D278 (green stick) and K324 (white stick) was detected a hydrogen bond during molecular dynamic analysis form a hydrogen bond. Residue E226 (orange sticks) is part of a helix (residues 221–231) connected to the loop that contains the catalytic acid-base residue E216 (pink stick).

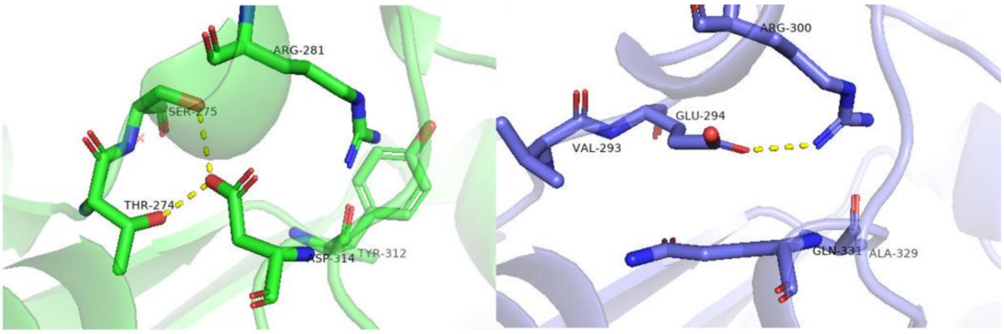

(a) (b)

Figure S10. Contact network of residue T274. (**a**) TmGTase and (**b**) its equivalent in Aspergillus oryzae α-amylase where T274 corresponds to V293 (PDB ID: 7taa).

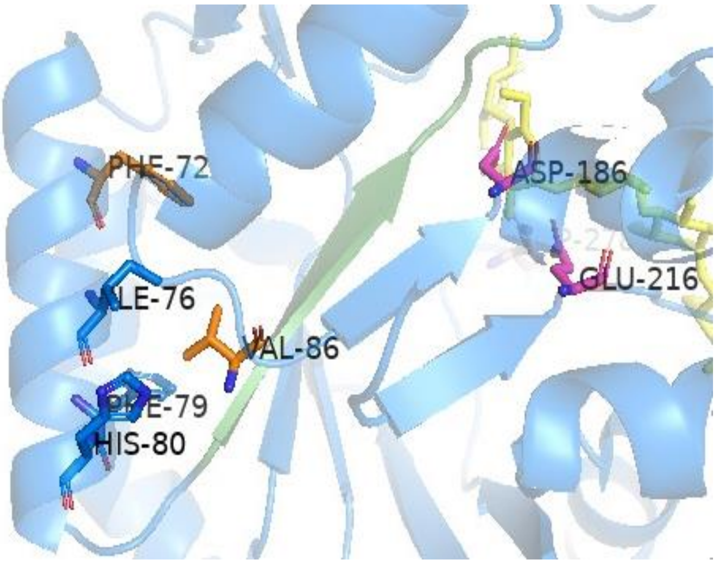

Figure S11. Residues F72 and V86 (orange sticks) affect the mobility and inclination of a β-strand (green cartoon) reaching the catalytic site of TmGTase. Positions 72 and 86 were additionally mutated in TmGTase. Modifying these residues far from the active site had a detrimental effect on activity, disfavoring the tranglycosidic activity preferentially. These residues interact indirectly with the active center through a β-strand constituted by residues 85 to 90, which form a super-secondary structure with the β-strands from 182–185 and 211–215, being in the last the acid-base residue.

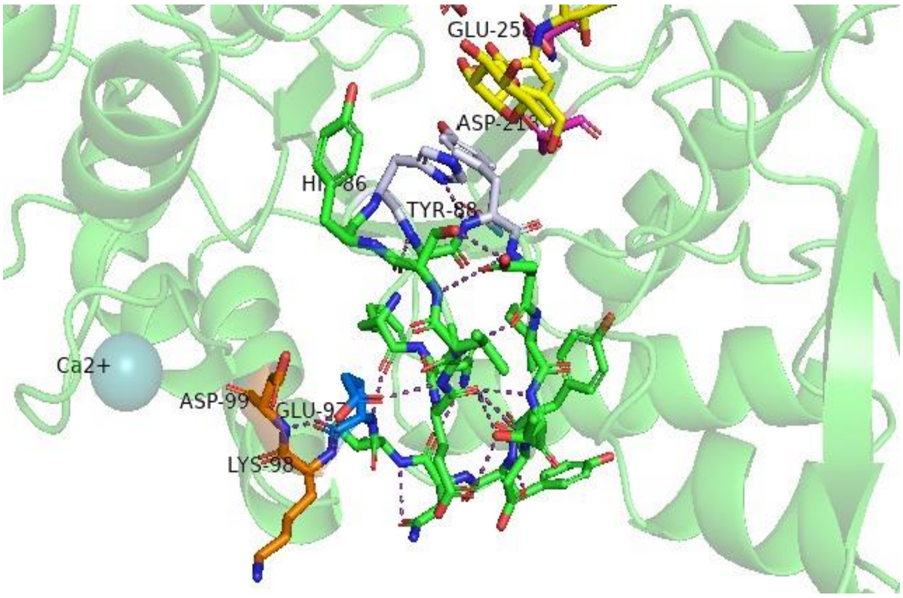

Figure S12. Residues K98 and D99 mediate the interaction of a calcium ion with the active site. A calcium ion (blueish green sphere) interacts with residues K98 and D99 (orange stick) in the TmAmyA 3D-structural model. Residues K98 and D99 connect the calcium ion to the +1 and +2 sites (H86, Y88) through a loop (green sticks). These sites are the acceptor binding positions during transglycosidation reaction. The inhibitor acarbose is shown in yellow sticks to show the binding subsites. The catalytic residues D218 and E258 (red and pink, respectively) delimit the enzyme's active center.

References

110. Wind, R.D.; Liebl, W.; Buitelaar, R.M.; Penninga, D.; Spreinat, A.; Dijkhuizen, L.; Bahl, H. Cyclodextrin formation by the thermostable alpha-amylase of Thermoanaerobacterium thermosulfurigenes EM1 and reclassification of the enzyme as a cyclodextrin glycosyltransferase. *Appl. Environ. Microbiol.* **1995**, *61*, 1257–1265, doi:10.1128/aem.61.4.1257-1265.1995.
111. Nakamura, A.; Haga, K.; Yamane, K. Four aromatic residues in the active center of cyclodextrin glucanotransferase from alkalophilic Bacillus sp. 1011: effects of replacements on substrate binding and cyclization characteristics. *Biochemistry* **1994**, *33*, 9929–9936, doi:10.1021/bi00199a015.
112. van der Veen, B.A.; van Alebeek, G.J.; Uitdehaag, J.C.; Dijkstra, B.W.; Dijkhuizen, L. The three transglycosylation reactions catalyzed by cyclodextrin glycosyltransferase from Bacillus circulans (strain 251) proceed via different kinetic mechanisms. *Eur. J. Biochem.* **2000**, *267*, 658–665, doi:10.1046/j.1432-1327.2000.01031.x.
113. Park, H.-S.; Park, J.-T.; Kang, H.-K.; Cha, H.; Kim, D.-S.; Kim, J.-W.; Park, K.-H. TreX from Sulfolobus solfataricus ATCC 35092 displays isoamylase and 4-alpha-glucanotransferase activities. *Biosci. Biotechnol. Biochem.* **2007**, *71*, 1348–1352, doi:10.1271/bbb.70016.
114. Jørgensen, S.; Vorgias, C.E.; Antranikian, G. Cloning, Sequencing, Characterization, and Expression of an Extracellular α-Amylase from the Hyperthermophilic ArchaeonPyrococcus furiosus in Escherichia coli andBacillus subtilis. *J. Biol. Chem.* **1997**, *272*, 16335–16342, doi:10.1074/jbc.272.26.16335.
115. Robyt, J.F.; French, D. The action pattern of porcine pancreatic alpha-amylase in relationship to the substrate binding site of the enzyme. *J. Biol. Chem.* **1970**, *245*, 3917–3927.
116. Kramhøft, B.; Bak-Jensen, K.S.; Mori, H.; Juge, N.; Nøhr, J.; Svensson, B. Involvement of individual subsites and secondary substrate binding sites in multiple attack on amylose by barley alpha-amylase. *Biochemistry* **2005**, *44*, 1824–1832, doi:10.1021/bi048100v.
117. Tonozuka, T.; Ohtsuka, M.; Mogi, S.; Sakai, H.; Ohta, T.; Sakano, Y. A neopullulanase-type alpha-amylase gene from Thermoactinomyces vulgaris R-47. *Biosci. Biotechnol. Biochem.* **1993**, *57*, 395–401, doi:10.1271/bbb.57.395.
118. Kanai, R.; Haga, K.; Akiba, T.; Yamane, K.; Harata, K. Biochemical and crystallographic analyses of maltohexaose-producing amylase from alkalophilic Bacillus sp. 707. *Biochemistry* **2004**, *43*, 14047–14056, doi:10.1021/bi048489m.
119. Brayer, G.D.; Sidhu, G.; Maurus, R.; Rydberg, E.H.; Braun, C.; Wang, Y.; Nguyen, N.T.; Overall, C.M.; Withers, S.G. Subsite mapping of the human pancreatic alpha-amylase active site through structural, kinetic, and mutagenesis techniques. *Biochemistry* **2000**, *39*, 4778–4791, doi:10.1021/bi9921182.
120. Choi, J.-H.; Lee, H.; Kim, Y.-W.; Park, J.-T.; Woo, E.-J.; Kim, M.-J.; Lee, B.-H.; Park, K.-H. Characterization of a novel debranching enzyme from Nostoc punctiforme possessing a high specificity for long branched chains. *Biochem. Biophys. Res. Commun.* **2009**, *378*, 224–229, doi:10.1016/j.bbrc.2008.11.020.
121. Tan, T.-C.; Mijts, B.N.; Swaminathan, K.; Patel, B.K.C.; Divne, C. Crystal structure of the polyextremophilic alpha-amylase AmyB from Halothermothrix orenii: details of a productive enzyme-substrate complex and an N domain with a role in binding raw starch. *J. Mol. Biol.* **2008**, *378*, 852–870, doi:10.1016/j.jmb.2008.02.041.
122. Shipman, J.A.; Cho, K.H.; Siegel, H.A.; Salyers, A.A. Physiological characterization of SusG, an outer membrane protein essential for starch utilization by Bacteroides thetaiotaomicron. *J. Bacteriol.* **1999**, *181*, 7206–7211, doi:10.1128/JB.181.23.7206-7211.1999.
123. Nitta, Y.; Mizushima, M.; Hiromi, K.; Ono, S. Influence of molecular structures of substrates and analogues on Taka-amylase A catalyzed hydrolyses. I. Effect of chain length of linear substrates. *J. Biochem.* **1971**, *69*, 567–576.
124. Nitschke, L.; Heeger, K.; Bender, H.; Schulz, G.E. Molecular cloning, nucleotide sequence and expression in Escherichia coli of the beta-cyclodextrin glycosyltransferase gene from Bacillus circulans strain no. 8. *Appl. Microbiol. Biotechnol.* **1990**, *33*, 542–546, doi:10.1007/BF00172548.
125. Li, Z.; Li, B.; Gu, Z.; Du, G.; Wu, J.; Chen, J. Extracellular expression and biochemical characterization of alpha-cyclodextrin glycosyltransferase from Paenibacillus macerans. *Carbohydr. Res.* **2010**, *345*, 886–892, doi:10.1016/j.carres.2010.02.002.
126. Violet, M.; Meunier, J.C. Kinetic study of the irreversible thermal denaturation of Bacillus licheniformis alpha-amylase. *Biochem. J.* **1989**, *263*, 665–670, doi:10.1042/bj2630665.
127. Nakada, T.; Kubota, M.; Sakai, S.; Tsujisaka, Y. Purification and characterization of two forms of maltotetraose-forming amylase from Pseudomonas stutzeri. *Agric. Biol. Chem.* **1990**, *54*, 737–743.
128. Tonozuka, T.; Mogi, S.; Shimura, Y.; Ibuka, A.; Sakai, H.; Matsuzawa, H.; Sakano, Y.; Ohta, T. Comparison of primary structures and substrate specificities of two pullulan-hydrolyzing alpha-amylases, TVA I and TVA II, from Thermoactinomyces vulgaris R-47. *Biochim. Biophys. Acta* **1995**, *1252*, 35–42, doi:10.1016/0167-4838(95)00101-y.
129. Mijts, B.N.; Patel, B.K.C. Cloning, sequencing and expression of an alpha-amylase gene, amyA, from the thermophilic halophile Halothermothrix orenii and purification and biochemical characterization of the recombinant enzyme. *Microbiology* **2002**, *148*, 2343–2349, doi:10.1099/00221287-148-8-2343.

130. Plaza-Vinuesa, L.; Hernandez-Hernandez, O.; Moreno, F.J.; de Las Rivas, B.; Muñoz, R. Unravelling the diversity of glycoside hydrolase family 13 α-amylases from Lactobacillus plantarum WCFS1. *Microb. Cell Fact.* **2019**, *18*, 1-11, doi:10.1186/s12934-019-1237-3.
131. Buedenbender, S.; Schulz, G.E. Structural base for enzymatic cyclodextrin hydrolysis. *J. Mol. Biol.* **2009**, *385*, 606–617, doi:10.1016/j.jmb.2008.10.085.
132. Han, P.; Zhou, P.; Hu, S.; Yang, S.; Yan, Q.; Jiang, Z. A novel multifunctional α-amylase from the thermophilic fungus Malbranchea cinnamomea: biochemical characterization and three-dimensional structure. *Appl. Biochem. Biotechnol.* **2013**, *170*, 420–435, doi:10.1007/s12010-013-0198-y.
133. Li, D.; Park, J.-T.; Li, X.; Kim, S.; Lee, S.; Shim, J.-H.; Park, S.-H.; Cha, J.; Lee, B.-H.; Kim, J.-W.; et al. Overexpression and characterization of an extremely thermostable maltogenic amylase, with an optimal temperature of 100 degrees C, from the hyperthermophilic archaeon Staphylothermus marinus. *N. Biotechnol.* **2010**, *27*, 300–307, doi:10.1016/j.nbt.2010.04.001.
134. Park, J.-T.; Song, H.-N.; Jung, T.-Y.; Lee, M.-H.; Park, S.-G.; Woo, E.-J.; Park, K.-H. A novel domain arrangement in a monomeric cyclodextrin-hydrolyzing enzyme from the hyperthermophile Pyrococcus furiosus. *Biochim. Biophys. Acta* **2013**, *1834*, 380–386, doi:10.1016/j.bbapap.2012.08.001.
135. Jun, S.-Y.; Kim, J.-S.; Choi, K.-H.; Cha, J.; Ha, N.-C. Structure of a novel α-amylase AmyB from Thermotoga neapolitana that produces maltose from the nonreducing end of polysaccharides. *Acta Crystallogr. D. Biol. Crystallogr.* **2013**, *69*, 442–450, doi:10.1107/S0907444912049219.
136. Tomazic, S.J.; Klibanov, A.M. Mechanisms of irreversible thermal inactivation of Bacillus alpha-amylases. *J. Biol. Chem.* **1988**, *263*, 3086–3091.
